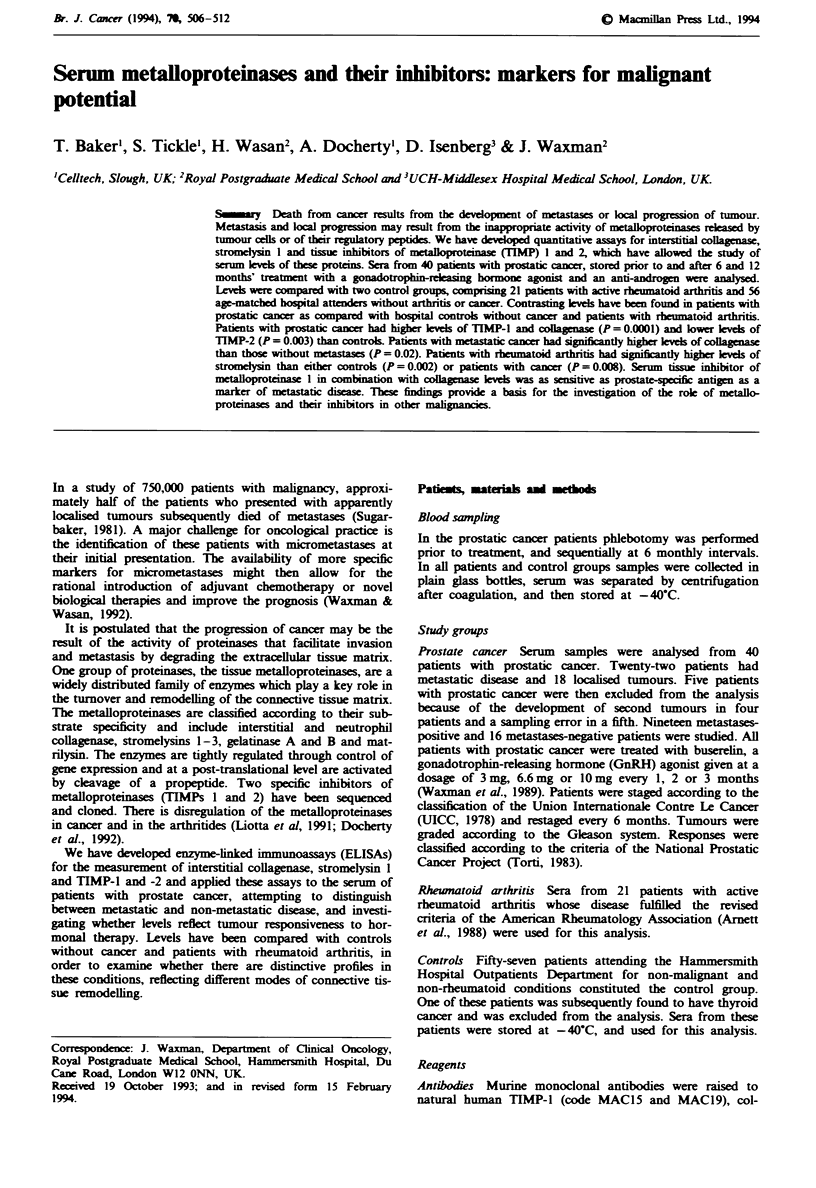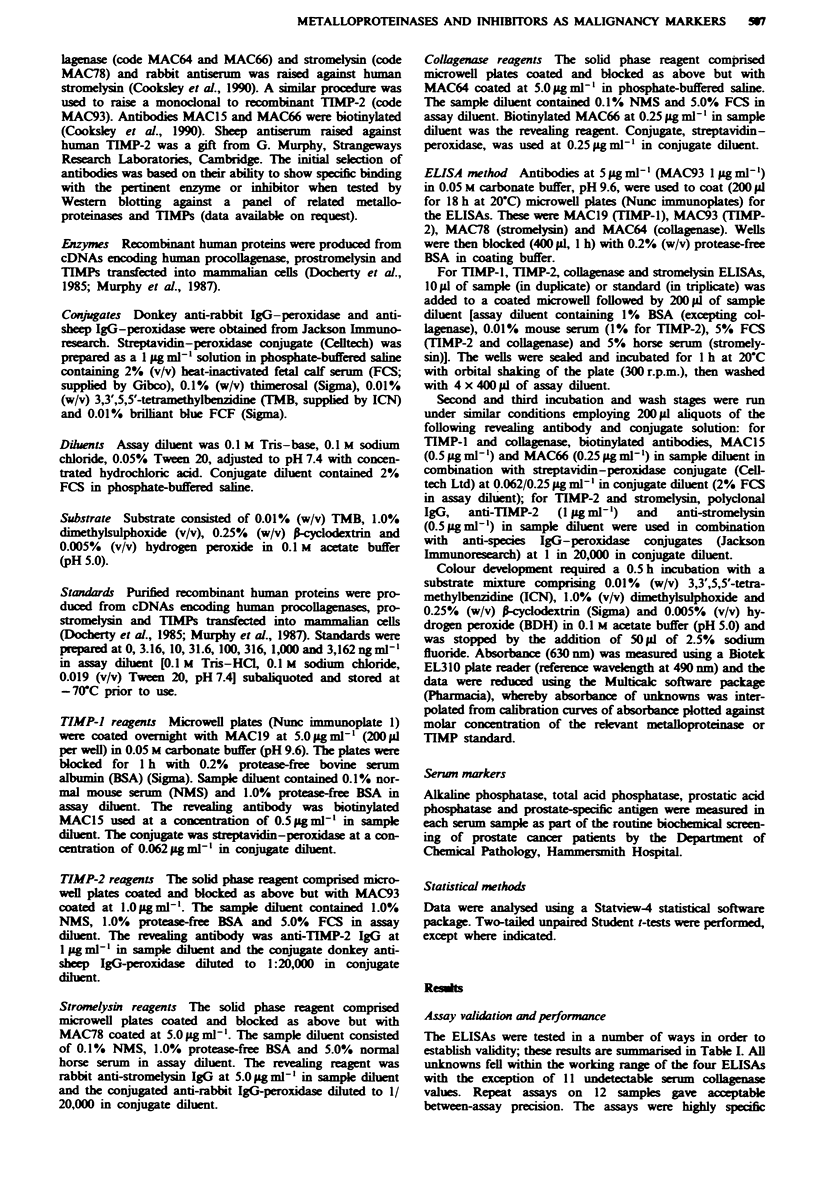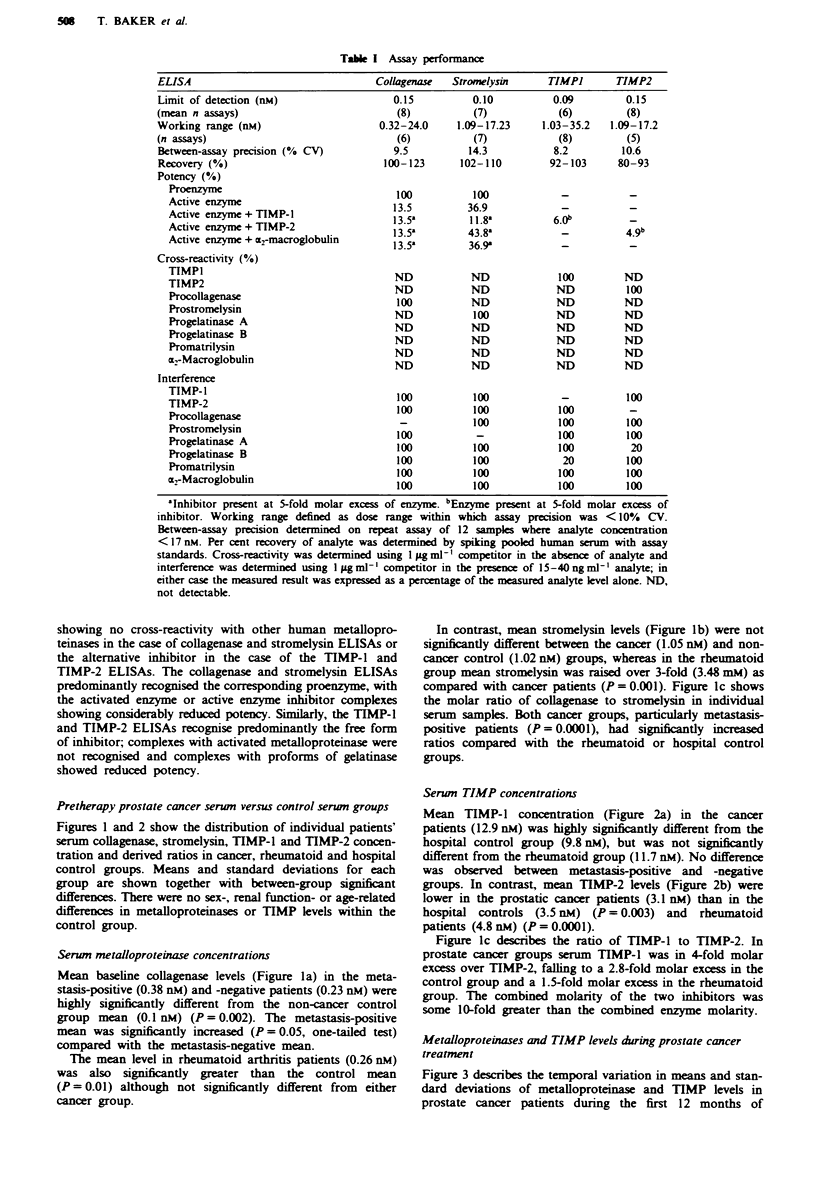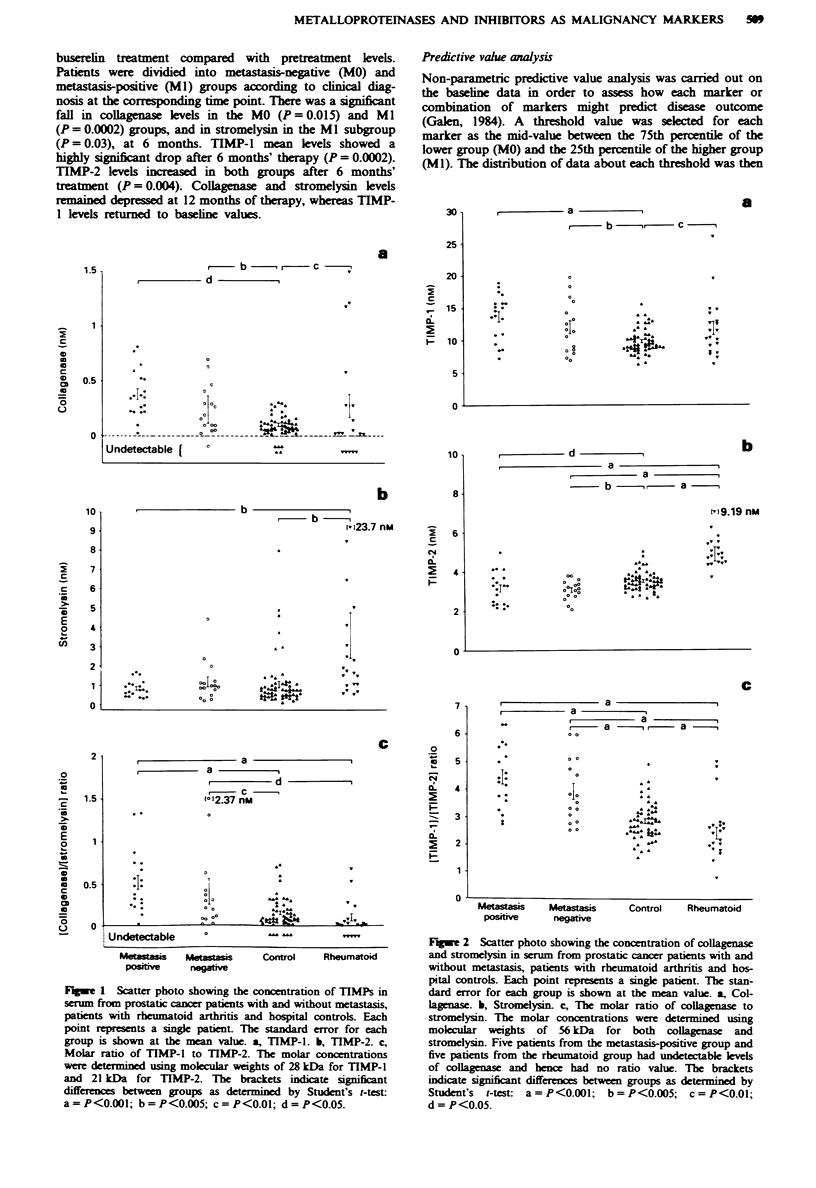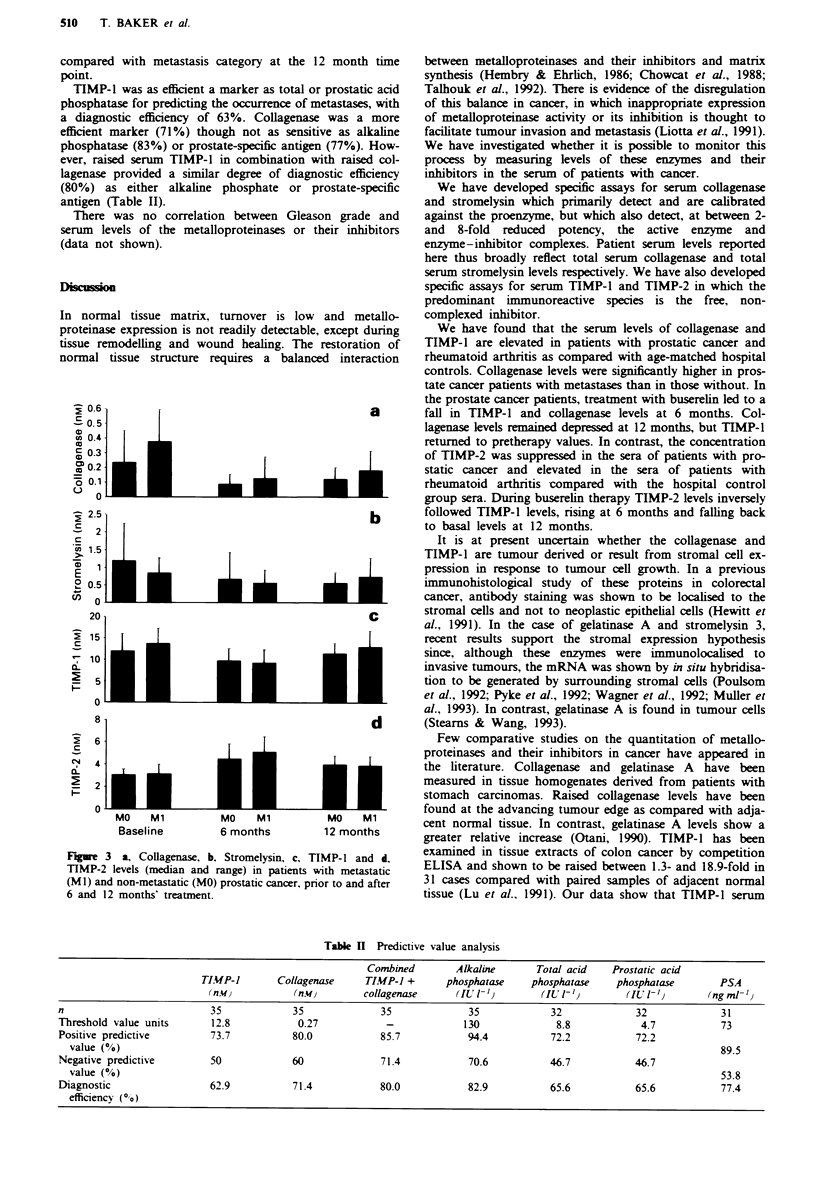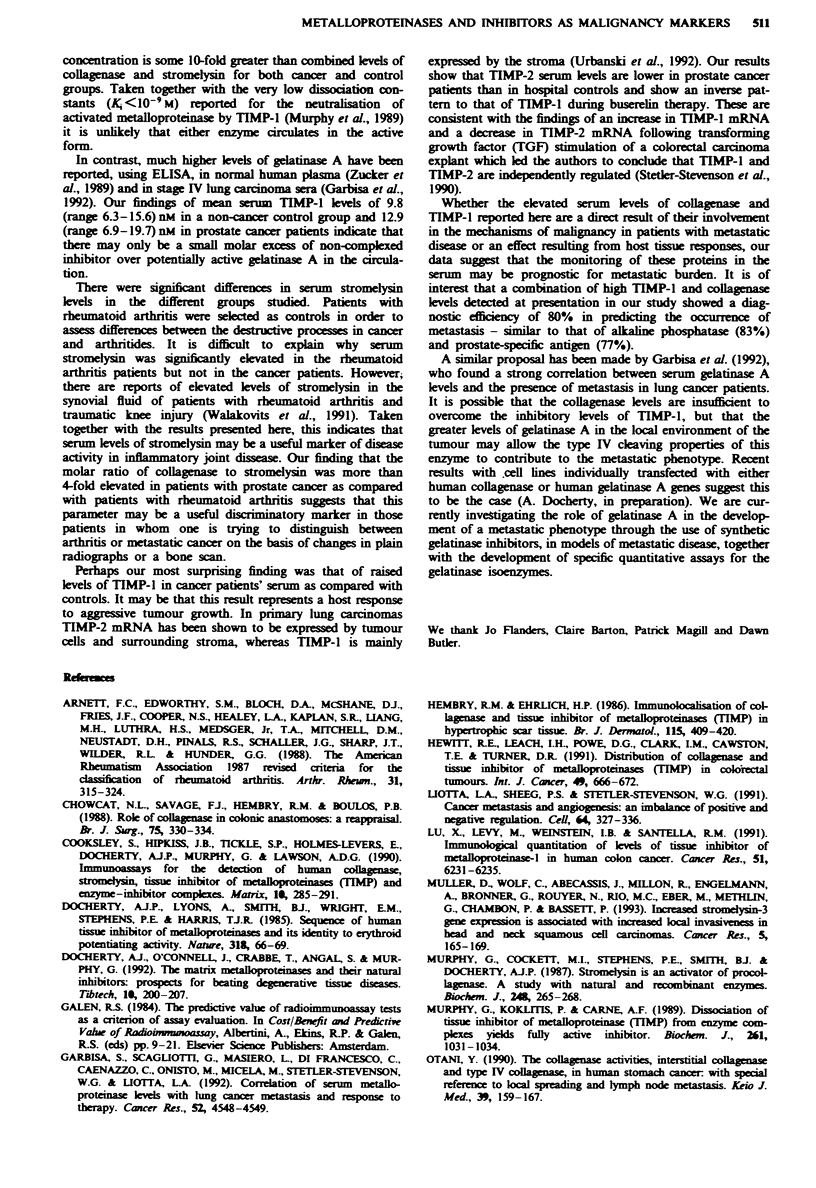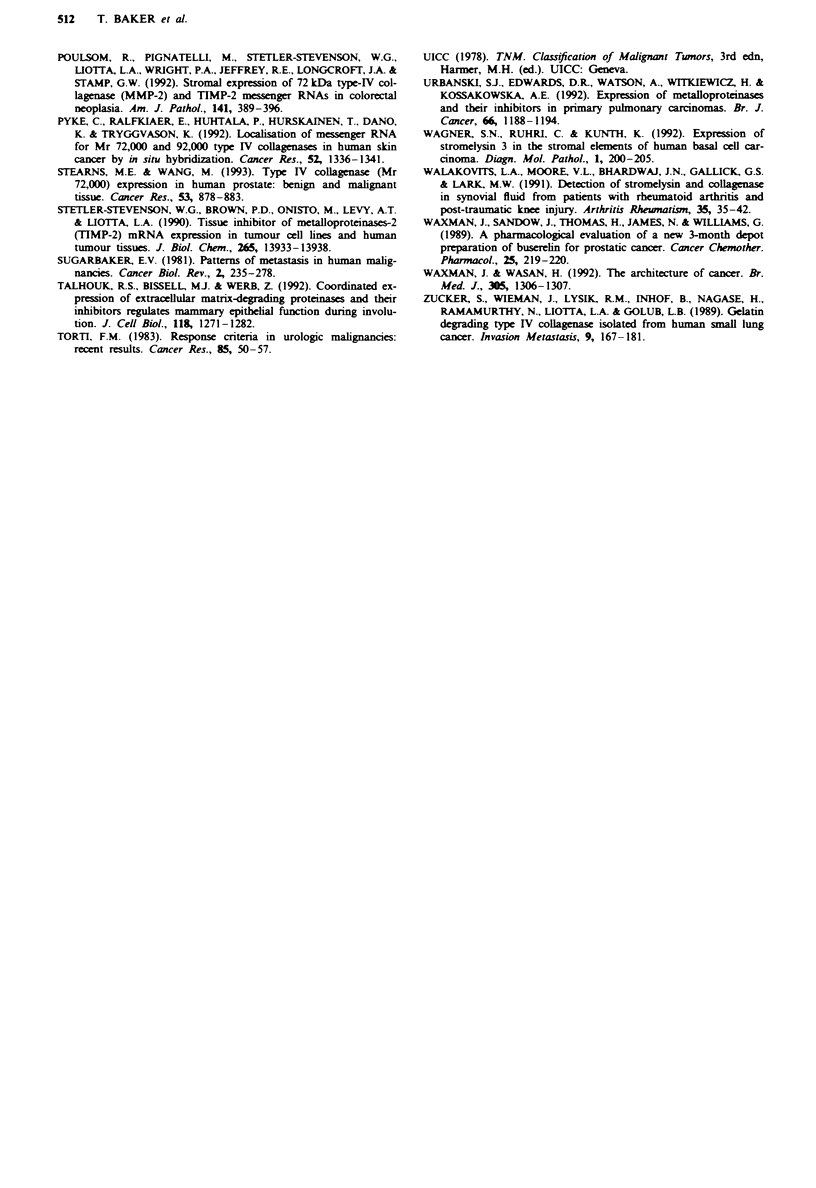# Serum metalloproteinases and their inhibitors: markers for malignant potential.

**DOI:** 10.1038/bjc.1994.336

**Published:** 1994-09

**Authors:** T. Baker, S. Tickle, H. Wasan, A. Docherty, D. Isenberg, J. Waxman

**Affiliations:** Celltech, Slough, UK.

## Abstract

Death from cancer results from the development of metastases or local progression of tumour. Metastasis and local progression may result from the inappropriate activity of metalloproteinases released by tumour cells or of their regulatory peptides. We have developed quantitative assays for interstitial collagenase, stromelysin 1 and tissue inhibitors of metalloproteinase (TIMP) 1 and 2, which have allowed the study of serum levels of these proteins. Sera from 40 patients with prostatic cancer, stored prior to and after 6 and 12 months' treatment with a gonadotrophin-releasing hormone agonist and an anti-androgen were analysed. Levels were compared with two control groups, comprising 21 patients with active rheumatoid arthritis and 56 age-matched hospital attenders without arthritis or cancer. Contrasting levels have been found in patients with prostatic cancer as compared with hospital controls without cancer and patients with rheumatoid arthritis. Patients with prostatic cancer had higher levels of TIMP-1 and collagenase (P = 0.0001) and lower levels of TIMP-2 (P = 0.003) than controls. Patients with metastatic cancer had significantly higher levels of collagenase than those without metastases (P = 0.02). Patients with rheumatoid arthritis had significantly higher levels of stromelysin than either controls (P = 0.002) or patients with cancer (P = 0.008). Serum tissue inhibitor of metalloproteinase 1 in combination with collagenase levels was as sensitive as prostate-specific antigen as a marker of metastatic disease. These findings provide a basis for the investigation of the role of metalloproteinases and their inhibitors in other malignancies.


 © Macmillan Press Ltd., 1994

### Serum metalloproteinases and their inhibitors: markers for malignant potential

T. Baker[1], S. Tickle[1], H. Wasan[2], A. Docherty[1], D. Isenberg[3] & J. Waxman[2]

[1]Celltech, Slough, UK; [2]Royal Postgraduate Medical School and [3]UCH-Middlesex Hospital Medical School, London, UK.

**Summary** Death from cancer results from the development of metastases or local progression of tumour. Metastasis and local progression may result from the inappropriate activity of metalloproteinases released by tumour cells or of their regulatory peptides. We have developed quantitative assays for interstitial collagenase, stromelysin 1 and tissue inhibitors of metalloproteinase (TIMP) 1 and 2, which have allowed the study of serum levels of these proteins. Sera from 40 patients with prostatic cancer, stored prior to and after 6 and 12 months' treatment with a gonadotrophin-releasing hormone agonist and an anti-androgen were analysed. Levels were compared with two control groups, comprising 21 patients with active rheumatoid arthritis and 56 age-matched hospital attenders without arthritis or cancer. Contrasting levels have been found in patients with prostatic cancer as compared with hospital controls without cancer and patients with rheumatoid arthritis. Patients with prostatic cancer had higher levels of TIMP-1 and collagenase ($P = 0.0001$) and lower levels of TIMP-2 ($P = 0.003$) than controls. Patients with metastatic cancer had significantly higher levels of collagenase than those without metastases ($P = 0.02$). Patients with rheumatoid arthritis had significantly higher levels of stromelysin than either controls ($P = 0.002$) or patients with cancer ($P = 0.008$). Serum tissue inhibitor of metalloproteinase 1 in combination with collagenase levels was as sensitive as prostate-specific antigen as a marker of metastatic disease. These findings provide a basis for the investigation of the role of metalloproteinases and their inhibitors in other malignancies.

In a study of 750,000 patients with malignancy, approximately half of the patients who presented with apparently localised tumours subsequently died of metastases (Sugarbaker, 1981). A major challenge for oncological practice is the identification of these patients with micrometastases at their initial presentation. The availability of more specific markers for micrometastases might then allow for the rational introduction of adjuvant chemotherapy or novel biological therapies and improve the prognosis (Waxman & Wasan, 1992).

It is postulated that the progression of cancer may be the result of the activity of proteinases that facilitate invasion and metastasis by degrading the extracellular tissue matrix. One group of proteinases, the tissue metalloproteinases, are a widely distributed family of enzymes which play a key role in the turnover and remodelling of the connective tissue matrix. The metalloproteinases are classified according to their substrate specificity and include interstitial and neutrophil collagenase, stromelysins 1-3, gelatinase A and B and matrilysin. The enzymes are tightly regulated through control of gene expression and at a post-translational level are activated by cleavage of a propeptide. Two specific inhibitors of metalloproteinases (TIMPs 1 and 2) have been sequenced and cloned. There is disregulation of the metalloproteinases in cancer and in the arthritides (Liotta *et al*, 1991; Docherty *et al*., 1992).

We have developed enzyme-linked immunoassays (ELISAs) for the measurement of interstitial collagenase, stromelysin 1 and TIMP-1 and -2 and applied these assays to the serum of patients with prostate cancer, attempting to distinguish between metastatic and non-metastatic disease, and investigating whether levels reflect tumour responsiveness to hormonal therapy. Levels have been compared with controls without cancer and patients with rheumatoid arthritis, in order to examine whether there are distinctive profiles in these conditions, reflecting different modes of connective tissue remodelling.

Correspondence: J. Waxman, Department of Clinical Oncology, Royal Postgraduate Medical School, Hammersmith Hospital, Du Cane Road, London W12 0NN, UK.
Received 19 October 1993; and in revised form 15 February 1994.

### Patients, materials and methods

### Blood sampling

In the prostatic cancer patients phlebotomy was performed prior to treatment, and sequentially at 6 monthly intervals. In all patients and control groups samples were collected in plain glass bottles, serum was separated by centrifugation after coagulation, and then stored at −40°C.

### Study groups

*Prostate cancer* Serum samples were analysed from 40 patients with prostatic cancer. Twenty-two patients had metastatic disease and 18 localised tumours. Five patients with prostatic cancer were then excluded from the analysis because of the development of second tumours in four patients and a sampling error in a fifth. Nineteen metastases-positive and 16 metastases-negative patients were studied. All patients with prostatic cancer were treated with buserelin, a gonadotrophin-releasing hormone (GnRH) agonist given at a dosage of 3 mg, 6.6 mg or 10 mg every 1, 2 or 3 months (Waxman *et al*., 1989). Patients were staged according to the classification of the Union Internationale Contre Le Cancer (UICC, 1978) and restaged every 6 months. Tumours were graded according to the Gleason system. Responses were classified according to the criteria of the National Prostatic Cancer Project (Torti, 1983).

*Rheumatoid arthritis* Sera from 21 patients with active rheumatoid arthritis whose disease fulfilled the revised criteria of the American Rheumatology Association (Arnett *et al*., 1988) were used for this analysis.

*Controls* Fifty-seven patients attending the Hammersmith Hospital Outpatients Department for non-malignant and non-rheumatoid conditions constituted the control group. One of these patients was subsequently found to have thyroid cancer and was excluded from the analysis. Sera from these patients were stored at −40°C, and used for this analysis.

### Reagents

*Antibodies* Murine monoclonal antibodies were raised to natural human TIMP-1 (code MAC15 and MAC19), collagenase (code MAC64 and MAC66) and stromelysin (code MAC78) and rabbit antiserum was raised against human stromelysin (Cooksley *et al.*, 1990). A similar procedure was used to raise a monoclonal to recombinant TIMP-2 (code MAC93). Antibodies MAC15 and MAC66 were biotinylated (Cooksley *et al.*, 1990). Sheep antiserum raised against human TIMP-2 was a gift from G. Murphy, Strangeways Research Laboratories, Cambridge. The initial selection of antibodies was based on their ability to show specific binding with the pertinent enzyme or inhibitor when tested by Western blotting against a panel of related metalloproteinases and TIMPs (data available on request).

*Enzymes* Recombinant human proteins were produced from cDNAs encoding human procollagenase, prostromelysin and TIMPs transfected into mammalian cells (Docherty *et al.*, 1985; Murphy *et al.*, 1987).

*Conjugates* Donkey anti-rabbit IgG–peroxidase and anti-sheep IgG–peroxidase were obtained from Jackson Immunoresearch. Streptavidin–peroxidase conjugate (Celltech) was prepared as a 1 $\mu$g ml$^{-1}$ solution in phosphate-buffered saline containing 2% (v/v) heat-inactivated fetal calf serum (FCS; supplied by Gibco), 0.1% (w/v) thimerosal (Sigma), 0.01% (w/v) 3,3′,5,5′-tetramethylbenzidine (TMB, supplied by ICN) and 0.01% brilliant blue FCF (Sigma).

*Diluents* Assay diluent was 0.1 M Tris–base, 0.1 M sodium chloride, 0.05% Tween 20, adjusted to pH 7.4 with concentrated hydrochloric acid. Conjugate diluent contained 2% FCS in phosphate-buffered saline.

*Substrate* Substrate consisted of 0.01% (w/v) TMB, 1.0% dimethylsulphoxide (v/v), 0.25% (w/v) $\beta$-cyclodextrin and 0.005% (v/v) hydrogen peroxide in 0.1 M acetate buffer (pH 5.0).

*Standards* Purified recombinant human proteins were produced from cDNAs encoding human procollagenases, prostromelysin and TIMPs transfected into mammalian cells (Docherty *et al.*, 1985; Murphy *et al.*, 1987). Standards were prepared at 0, 3.16, 10, 31.6, 100, 316, 1,000 and 3,162 ng ml$^{-1}$ in assay diluent [0.1 M Tris–HCl, 0.1 M sodium chloride, 0.019 (v/v) Tween 20, pH 7.4] subaliquoted and stored at −70°C prior to use.

*TIMP-1 reagents* Microwell plates (Nunc immunoplate 1) were coated overnight with MAC19 at 5.0 $\mu$g ml$^{-1}$ (200 $\mu$l per well) in 0.05 M carbonate buffer (pH 9.6). The plates were blocked for 1 h with 0.2% protease-free bovine serum albumin (BSA) (Sigma). Sample diluent contained 0.1% normal mouse serum (NMS) and 1.0% protease-free BSA in assay diluent. The revealing antibody was biotinylated MAC15 used at a concentration of 0.5 $\mu$g ml$^{-1}$ in sample diluent. The conjugate was streptavidin–peroxidase at a concentration of 0.062 $\mu$g ml$^{-1}$ in conjugate diluent.

*TIMP-2 reagents* The solid phase reagent comprised microwell plates coated and blocked as above but with MAC93 coated at 1.0 $\mu$g ml$^{-1}$. The sample diluent contained 1.0% NMS, 1.0% protease-free BSA and 5.0% FCS in assay diluent. The revealing antibody was anti-TIMP-2 IgG at 1 $\mu$g ml$^{-1}$ in sample diluent and the conjugate donkey anti-sheep IgG-peroxidase diluted to 1:20,000 in conjugate diluent.

*Stromelysin reagents* The solid phase reagent comprised microwell plates coated and blocked as above but with MAC78 coated at 5.0 $\mu$g ml$^{-1}$. The sample diluent consisted of 0.1% NMS, 1.0% protease-free BSA and 5.0% normal horse serum in assay diluent. The revealing reagent was rabbit anti-stromelysin IgG at 5.0 $\mu$g ml$^{-1}$ in sample diluent and the conjugated anti-rabbit IgG-peroxidase diluted to 1/20,000 in conjugate diluent.

*Collagenase reagents* The solid phase reagent comprised microwell plates coated and blocked as above but with MAC64 coated at 5.0 $\mu$g ml$^{-1}$ in phosphate-buffered saline. The sample diluent contained 0.1% NMS and 5.0% FCS in assay diluent. Biotinylated MAC66 at 0.25 $\mu$g ml$^{-1}$ in sample diluent was the revealing reagent. Conjugate, streptavidin–peroxidase, was used at 0.25 $\mu$g ml$^{-1}$ in conjugate diluent.

*ELISA method* Antibodies at 5 $\mu$g ml$^{-1}$ (MAC93 1 $\mu$g ml$^{-1}$) in 0.05 M carbonate buffer, pH 9.6, were used to coat (200 $\mu$l for 18 h at 20°C) microwell plates (Nunc immunoplates) for the ELISAs. These were MAC19 (TIMP-1), MAC93 (TIMP-2), MAC78 (stromelysin) and MAC64 (collagenase). Wells were then blocked (400 $\mu$l, 1 h) with 0.2% (w/v) protease-free BSA in coating buffer.

For TIMP-1, TIMP-2, collagenase and stromelysin ELISAs, 10 $\mu$l of sample (in duplicate) or standard (in triplicate) was added to a coated microwell followed by 200 $\mu$l of sample diluent [assay diluent containing 1% BSA (excepting collagenase), 0.01% mouse serum (1% for TIMP-2), 5% FCS (TIMP-2 and collagenase) and 5% horse serum (stromelysin)]. The wells were sealed and incubated for 1 h at 20°C with orbital shaking of the plate (300 r.p.m.), then washed with 4 × 400 $\mu$l of assay diluent.

Second and third incubation and wash stages were run under similar conditions employing 200 $\mu$l aliquots of the following revealing antibody and conjugate solution: for TIMP-1 and collagenase, biotinylated antibodies, MAC15 (0.5 $\mu$g ml$^{-1}$) and MAC66 (0.25 $\mu$g ml$^{-1}$) in sample diluent in combination with streptavidin–peroxidase conjugate (Celltech Ltd) at 0.062/0.25 $\mu$g ml$^{-1}$ in conjugate diluent (2% FCS in assay diluent); for TIMP-2 and stromelysin, polyclonal IgG, anti-TIMP-2 (1 $\mu$g ml$^{-1}$) and anti-stromelysin (0.5 $\mu$g ml$^{-1}$) in sample diluent were used in combination with anti-species IgG–peroxidase conjugates (Jackson Immunoresearch) at 1 in 20,000 in conjugate diluent.

Colour development required a 0.5 h incubation with a substrate mixture comprising 0.01% (w/v) 3,3′,5,5′-tetramethylbenzidine (ICN), 1.0% (v/v) dimethylsulphoxide and 0.25% (w/v) $\beta$-cyclodextrin (Sigma) and 0.005% (v/v) hydrogen peroxide (BDH) in 0.1 M acetate buffer (pH 5.0) and was stopped by the addition of 50 $\mu$l of 2.5% sodium fluoride. Absorbance (630 nm) was measured using a Biotek EL310 plate reader (reference wavelength at 490 nm) and the data were reduced using the Multicalc software package (Pharmacia), whereby absorbance of unknowns was interpolated from calibration curves of absorbance plotted against molar concentration of the relevant metalloproteinase or TIMP standard.

### *Serum markers*

Alkaline phosphatase, total acid phosphatase, prostatic acid phosphatase and prostate-specific antigen were measured in each serum sample as part of the routine biochemical screening of prostate cancer patients by the Department of Chemical Pathology, Hammersmith Hospital.

### *Statistical methods*

Data were analysed using a Statview-4 statistical software package. Two-tailed unpaired Student *t*-tests were performed, except where indicated.

### Results

### *Assay validation and performance*

The ELISAs were tested in a number of ways in order to establish validity; these results are summarised in Table I. All unknowns fell within the working range of the four ELISAs with the exception of 11 undetectable serum collagenase values. Repeat assays on 12 samples gave acceptable between-assay precision. The assays were highly specific

**Table I** Assay performance

| *ELISA* | *Collagenase* | *Stromelysin* | *TIMP1* | *TIMP2* |
|---|---|---|---|---|
| Limit of detection (nM) | 0.15 | 0.10 | 0.09 | 0.15 |
| (mean *n* assays) | (8) | (7) | (6) | (8) |
| Working range (nM) | 0.32–24.0 | 1.09–17.23 | 1.03–35.2 | 1.09–17.2 |
| (*n* assays) | (6) | (7) | (8) | (5) |
| Between-assay precision (% CV) | 9.5 | 14.3 | 8.2 | 10.6 |
| Recovery (%) | 100–123 | 102–110 | 92–103 | 80–93 |
| Potency (%) | | | | |
| Proenzyme | 100 | 100 | – | – |
| Active enzyme | 13.5 | 36.9 | – | – |
| Active enzyme + TIMP-1 | 13.5[a] | 11.8[a] | 6.0[b] | – |
| Active enzyme + TIMP-2 | 13.5[a] | 43.8[a] | – | 4.9[b] |
| Active enzyme + $\alpha_2$-macroglobulin | 13.5[a] | 36.9[a] | – | – |
| Cross-reactivity (%) | | | | |
| TIMP1 | ND | ND | 100 | ND |
| TIMP2 | ND | ND | ND | 100 |
| Procollagenase | 100 | ND | ND | ND |
| Prostromelysin | ND | 100 | ND | ND |
| Progelatinase A | ND | ND | ND | ND |
| Progelatinase B | ND | ND | ND | ND |
| Promatrilysin | ND | ND | ND | ND |
| $\alpha_2$-Macroglobulin | ND | ND | ND | ND |
| Interference | | | | |
| TIMP-1 | 100 | 100 | – | 100 |
| TIMP-2 | 100 | 100 | 100 | – |
| Procollagenase | – | 100 | 100 | 100 |
| Prostromelysin | 100 | – | 100 | 100 |
| Progelatinase A | 100 | 100 | 100 | 20 |
| Progelatinase B | 100 | 100 | 20 | 100 |
| Promatrilysin | 100 | 100 | 100 | 100 |
| $\alpha_2$-Macroglobulin | 100 | 100 | 100 | 100 |

[a]Inhibitor present at 5-fold molar excess of enzyme. [b]Enzyme present at 5-fold molar excess of inhibitor. Working range defined as dose range within which assay precision was <10% CV. Between-assay precision determined on repeat assay of 12 samples where analyte concentration <17 nM. Per cent recovery of analyte was determined by spiking pooled human serum with assay standards. Cross-reactivity was determined using 1 μg $ml^{-1}$ competitor in the absence of analyte and interference was determined using 1 μg $ml^{-1}$ competitor in the presence of 15–40 ng $ml^{-1}$ analyte; in either case the measured result was expressed as a percentage of the measured analyte level alone. ND, not detectable.

showing no cross-reactivity with other human metalloproteinases in the case of collagenase and stromelysin ELISAs or the alternative inhibitor in the case of the TIMP-1 and TIMP-2 ELISAs. The collagenase and stromelysin ELISAs predominantly recognised the corresponding proenzyme, with the activated enzyme or active enzyme inhibitor complexes showing considerably reduced potency. Similarly, the TIMP-1 and TIMP-2 ELISAs recognise predominantly the free form of inhibitor; complexes with activated metalloproteinase were not recognised and complexes with proforms of gelatinase showed reduced potency.

### *Pretherapy prostate cancer serum versus control serum groups*

Figures 1 and 2 show the distribution of individual patients' serum collagenase, stromelysin, TIMP-1 and TIMP-2 concentration and derived ratios in cancer, rheumatoid and hospital control groups. Means and standard deviations for each group are shown together with between-group significant differences. There were no sex-, renal function- or age-related differences in metalloproteinases or TIMP levels within the control group.

### *Serum metalloproteinase concentrations*

Mean baseline collagenase levels (Figure 1a) in the metastasis-positive (0.38 nM) and -negative patients (0.23 nM) were highly significantly different from the non-cancer control group mean (0.1 nM) ($P = 0.002$). The metastasis-positive mean was significantly increased ($P = 0.05$, one-tailed test) compared with the metastasis-negative mean.

The mean level in rheumatoid arthritis patients (0.26 nM) was also significantly greater than the control mean ($P = 0.01$) although not significantly different from either cancer group.

In contrast, mean stromelysin levels (Figure 1b) were not significantly different between the cancer (1.05 nM) and non-cancer control (1.02 nM) groups, whereas in the rheumatoid group mean stromelysin was raised over 3-fold (3.48 mM) as compared with cancer patients ($P = 0.001$). Figure 1c shows the molar ratio of collagenase to stromelysin in individual serum samples. Both cancer groups, particularly metastasis-positive patients ($P = 0.0001$), had significantly increased ratios compared with the rheumatoid or hospital control groups.

### *Serum TIMP concentrations*

Mean TIMP-1 concentration (Figure 2a) in the cancer patients (12.9 nM) was highly significantly different from the hospital control group (9.8 nM), but was not significantly different from the rheumatoid group (11.7 nM). No difference was observed between metastasis-positive and -negative groups. In contrast, mean TIMP-2 levels (Figure 2b) were lower in the prostatic cancer patients (3.1 nM) than in the hospital controls (3.5 nM) ($P = 0.003$) and rheumatoid patients (4.8 nM) ($P = 0.0001$).

Figure 1c describes the ratio of TIMP-1 to TIMP-2. In prostate cancer groups serum TIMP-1 was in 4-fold molar excess over TIMP-2, falling to a 2.8-fold molar excess in the control group and a 1.5-fold molar excess in the rheumatoid group. The combined molarity of the two inhibitors was some 10-fold greater than the combined enzyme molarity.

### *Metalloproteinases and TIMP levels during prostate cancer treatment*

Figure 3 describes the temporal variation in means and standard deviations of metalloproteinase and TIMP levels in prostate cancer patients during the first 12 months of buserelin treatment compared with pretreatment levels. Patients were dividied into metastasis-negative (M0) and metastasis-positive (M1) groups according to clinical diagnosis at the corresponding time point. There was a significant fall in collagenase levels in the M0 ($P = 0.015$) and M1 ($P = 0.0002$) groups, and in stromelysin in the M1 subgroup ($P = 0.03$), at 6 months. TIMP-1 mean levels showed a highly significant drop after 6 months' therapy ($P = 0.0002$). TIMP-2 levels increased in both groups after 6 months' treatment ($P = 0.004$). Collagenase and stromelysin levels remained depressed at 12 months of therapy, whereas TIMP-1 levels returned to baseline values.

**Figure 1** Scatter photo showing the concentration of TIMPs in serum from prostatic cancer patients with and without metastasis, patients with rheumatoid arthritis and hospital controls. Each point represents a single patient. The standard error for each group is shown at the mean value. **a**, TIMP-1. **b**, TIMP-2. **c**, Molar ratio of TIMP-1 to TIMP-2. The molar concentrations were determined using molecular weights of 28 kDa for TIMP-1 and 21 kDa for TIMP-2. The brackets indicate significant differences between groups as determined by Student's *t*-test: a = $P<0.001$; b = $P<0.005$; c = $P<0.01$; d = $P<0.05$.

### *Predictive value analysis*

Non-parametric predictive value analysis was carried out on the baseline data in order to assess how each marker or combination of markers might predict disease outcome (Galen, 1984). A threshold value was selected for each marker as the mid-value between the 75th percentile of the lower group (M0) and the 25th percentile of the higher group (M1). The distribution of data about each threshold was then

**Figure 2** Scatter photo showing the concentration of collagenase and stromelysin in serum from prostatic cancer patients with and without metastasis, patients with rheumatoid arthritis and hospital controls. Each point represents a single patient. The standard error for each group is shown at the mean value. **a**, Collagenase. **b**, Stromelysin. **c**, The molar ratio of collagenase to stromelysin. The molar concentrations were determined using molecular weights of 56 kDa for both collagenase and stromelysin. Five patients from the metastasis-positive group and five patients from the rheumatoid group had undetectable levels of collagenase and hence had no ratio value. The brackets indicate significant differences between groups as determined by Student's *t*-test: a = $P<0.001$; b = $P<0.005$; c = $P<0.01$; d = $P<0.05$.

compared with metastasis category at the 12 month time point.

TIMP-1 was as efficient a marker as total or prostatic acid phosphatase for predicting the occurrence of metastases, with a diagnostic efficiency of 63%. Collagenase was a more efficient marker (71%) though not as sensitive as alkaline phosphatase (83%) or prostate-specific antigen (77%). However, raised serum TIMP-1 in combination with raised collagenase provided a similar degree of diagnostic efficiency (80%) as either alkaline phosphate or prostate-specific antigen (Table II).

There was no correlation between Gleason grade and serum levels of the metalloproteinases or their inhibitors (data not shown).

### Discussion

In normal tissue matrix, turnover is low and metalloproteinase expression is not readily detectable, except during tissue remodelling and wound healing. The restoration of normal tissue structure requires a balanced interaction

**Figure 3** **a**, Collagenase, **b**, Stromelysin, **c**, TIMP-1 and **d**, TIMP-2 levels (median and range) in patients with metastatic (M1) and non-metastatic (M0) prostatic cancer, prior to and after 6 and 12 months' treatment.

between metalloproteinases and their inhibitors and matrix synthesis (Hembry & Ehrlich, 1986; Chowcat et al., 1988; Talhouk et al., 1992). There is evidence of the disregulation of this balance in cancer, in which inappropriate expression of metalloproteinase activity or its inhibition is thought to facilitate tumour invasion and metastasis (Liotta et al., 1991). We have investigated whether it is possible to monitor this process by measuring levels of these enzymes and their inhibitors in the serum of patients with cancer.

We have developed specific assays for serum collagenase and stromelysin which primarily detect and are calibrated against the proenzyme, but which also detect, at between 2- and 8-fold reduced potency, the active enzyme and enzyme-inhibitor complexes. Patient serum levels reported here thus broadly reflect total serum collagenase and total serum stromelysin levels respectively. We have also developed specific assays for serum TIMP-1 and TIMP-2 in which the predominant immunoreactive species is the free, non-complexed inhibitor.

We have found that the serum levels of collagenase and TIMP-1 are elevated in patients with prostatic cancer and rheumatoid arthritis as compared with age-matched hospital controls. Collagenase levels were significantly higher in prostate cancer patients with metastases than in those without. In the prostate cancer patients, treatment with buserelin led to a fall in TIMP-1 and collagenase levels at 6 months. Collagenase levels remained depressed at 12 months, but TIMP-1 returned to pretherapy values. In contrast, the concentration of TIMP-2 was suppressed in the sera of patients with prostatic cancer and elevated in the sera of patients with rheumatoid arthritis compared with the hospital control group sera. During buserelin therapy TIMP-2 levels inversely followed TIMP-1 levels, rising at 6 months and falling back to basal levels at 12 months.

It is at present uncertain whether the collagenase and TIMP-1 are tumour derived or result from stromal cell expression in response to tumour cell growth. In a previous immunohistological study of these proteins in colorectal cancer, antibody staining was shown to be localised to the stromal cells and not to neoplastic epithelial cells (Hewitt et al., 1991). In the case of gelatinase A and stromelysin 3, recent results support the stromal expression hypothesis since, although these enzymes were immunolocalised to invasive tumours, the mRNA was shown by *in situ* hybridisation to be generated by surrounding stromal cells (Poulsom et al., 1992; Pyke et al., 1992; Wagner et al., 1992; Muller et al., 1993). In contrast, gelatinase A is found in tumour cells (Stearns & Wang, 1993).

Few comparative studies on the quantitation of metalloproteinases and their inhibitors in cancer have appeared in the literature. Collagenase and gelatinase A have been measured in tissue homogenates derived from patients with stomach carcinomas. Raised collagenase levels have been found at the advancing tumour edge as compared with adjacent normal tissue. In contrast, gelatinase A levels show a greater relative increase (Otani, 1990). TIMP-1 has been examined in tissue extracts of colon cancer by competition ELISA and shown to be raised between 1.3- and 18.9-fold in 31 cases compared with paired samples of adjacent normal tissue (Lu et al., 1991). Our data show that TIMP-1 serum

**Table II** Predictive value analysis

| | *TIMP-1* (*nM*) | *Collagenase* (*nM*) | *Combined TIMP-1 + collagenase* | *Alkaline phosphatase* (*IU $l^{-1}$*) | *Total acid phosphatase* (*IU $l^{-1}$*) | *Prostatic acid phosphatase* (*IU $l^{-1}$*) | *PSA* (*ng $ml^{-1}$*) |
|---|---|---|---|---|---|---|---|
| *n* | 35 | 35 | 35 | 35 | 32 | 32 | 31 |
| Threshold value units | 12.8 | 0.27 | – | 130 | 8.8 | 4.7 | 73 |
| Positive predictive value (%) | 73.7 | 80.0 | 85.7 | 94.4 | 72.2 | 72.2 | 89.5 |
| Negative predictive value (%) | 50 | 60 | 71.4 | 70.6 | 46.7 | 46.7 | 53.8 |
| Diagnostic efficiency (%) | 62.9 | 71.4 | 80.0 | 82.9 | 65.6 | 65.6 | 77.4 |

concentration is some 10-fold greater than combined levels of collagenase and stromelysin for both cancer and control groups. Taken together with the very low dissociation constants ($K_i<10^{-9}$ M) reported for the neutralisation of activated metalloproteinase by TIMP-1 (Murphy *et al.*, 1989) it is unlikely that either enzyme circulates in the active form.

In contrast, much higher levels of gelatinase A have been reported, using ELISA, in normal human plasma (Zucker *et al.*, 1989) and in stage IV lung carcinoma sera (Garbisa *et al.*, 1992). Our findings of mean serum TIMP-1 levels of 9.8 (range 6.3–15.6) nM in a non-cancer control group and 12.9 (range 6.9–19.7) nM in prostate cancer patients indicate that there may only be a small molar excess of non-complexed inhibitor over potentially active gelatinase A in the circulation.

There were significant differences in serum stromelysin levels in the different groups studied. Patients with rheumatoid arthritis were selected as controls in order to assess differences between the destructive processes in cancer and arthritides. It is difficult to explain why serum stromelysin was significantly elevated in the rheumatoid arthritis patients but not in the cancer patients. However, there are reports of elevated levels of stromelysin in the synovial fluid of patients with rheumatoid arthritis and traumatic knee injury (Walakovits *et al.*, 1991). Taken together with the results presented here, this indicates that serum levels of stromelysin may be a useful marker of disease activity in inflammatory joint dissease. Our finding that the molar ratio of collagenase to stromelysin was more than 4-fold elevated in patients with prostate cancer as compared with patients with rheumatoid arthritis suggests that this parameter may be a useful discriminatory marker in those patients in whom one is trying to distinguish between arthritis or metastatic cancer on the basis of changes in plain radiographs or a bone scan.

Perhaps our most surprising finding was that of raised levels of TIMP-1 in cancer patients' serum as compared with controls. It may be that this result represents a host response to aggressive tumour growth. In primary lung carcinomas TIMP-2 mRNA has been shown to be expressed by tumour cells and surrounding stroma, whereas TIMP-1 is mainly expressed by the stroma (Urbanski *et al.*, 1992). Our results show that TIMP-2 serum levels are lower in prostate cancer patients than in hospital controls and show an inverse pattern to that of TIMP-1 during buserelin therapy. These are consistent with the findings of an increase in TIMP-1 mRNA and a decrease in TIMP-2 mRNA following transforming growth factor (TGF) stimulation of a colorectal carcinoma explant which led the authors to conclude that TIMP-1 and TIMP-2 are independently regulated (Stetler-Stevenson *et al.*, 1990).

Whether the elevated serum levels of collagenase and TIMP-1 reported here are a direct result of their involvement in the mechanisms of malignancy in patients with metastatic disease or an effect resulting from host tissue responses, our data suggest that the monitoring of these proteins in the serum may be prognostic for metastatic burden. It is of interest that a combination of high TIMP-1 and collagenase levels detected at presentation in our study showed a diagnostic efficiency of 80% in predicting the occurrence of metastasis – similar to that of alkaline phosphatase (83%) and prostate-specific antigen (77%).

A similar proposal has been made by Garbisa *et al.* (1992), who found a strong correlation between serum gelatinase A levels and the presence of metastasis in lung cancer patients. It is possible that the collagenase levels are insufficient to overcome the inhibitory levels of TIMP-1, but that the greater levels of gelatinase A in the local environment of the tumour may allow the type IV cleaving properties of this enzyme to contribute to the metastatic phenotype. Recent results with cell lines individually transfected with either human collagenase or human gelatinase A genes suggest this to be the case (A. Docherty, in preparation). We are currently investigating the role of gelatinase A in the development of a metastatic phenotype through the use of synthetic gelatinase inhibitors, in models of metastatic disease, together with the development of specific quantitative assays for the gelatinase isoenzymes.

We thank Jo Flanders, Claire Barton, Patrick Magill and Dawn Butler.

### References

ARNETT, F.C., EDWORTHY, S.M., BLOCH, D.A., MCSHANE, D.J., FRIES, J.F., COOPER, N.S., HEALEY, L.A., KAPLAN, S.R., LIANG, M.H., LUTHRA, H.S., MEDSGER, Jr, T.A., MITCHELL, D.M., NEUSTADT, D.H., PINALS, R.S., SCHALLER, J.G., SHARP, J.T., WILDER, R.L. & HUNDER, G.G. (1988). The American Rheumatism Association 1987 revised criteria for the classification of rheumatoid arthritis. *Arthr. Rheum.*, **31**, 315–324.

CHOWCAT, N.L., SAVAGE, F.J., HEMBRY, R.M. & BOULOS, P.B. (1988). Role of collagenase in colonic anastomoses: a reappraisal. *Br. J. Surg.*, **75**, 330–334.

COOKSLEY, S., HIPKISS, J.B., TICKLE, S.P., HOLMES-LEVERS, E., DOCHERTY, A.J.P., MURPHY, G. & LAWSON, A.D.G. (1990). Immunoassays for the detection of human collagenase, stromelysin, tissue inhibitor of metalloproteinases (TIMP) and enzyme-inhibitor complexes. *Matrix*, **10**, 285–291.

DOCHERTY, A.J.P., LYONS, A., SMITH, B.J., WRIGHT, E.M., STEPHENS, P.E. & HARRIS, T.J.R. (1985). Sequence of human tissue inhibitor of metalloproteinases and its identity to erythroid potentiating activity. *Nature*, **318**, 66–69.

DOCHERTY, A.J., O'CONNELL, J., CRABBE, T., ANGAL, S. & MURPHY, G. (1992). The matrix metalloproteinases and their natural inhibitors: prospects for beating degenerative tissue diseases. *Tibtech*, **10**, 200–207.

GALEN, R.S. (1984). The predictive value of radioimmunoassay tests as a criterion of assay evaluation. In *Cost/Benefit and Predictive Value of Radioimmunoassay*, Albertini, A., Ekins, R.P. & Galen, R.S. (eds) pp. 9–21. Elsevier Science Publishers: Amsterdam.

GARBISA, S., SCAGLIOTTI, G., MASIERO, L., DI FRANCESCO, C., CAENAZZO, C., ONISTO, M., MICELA, M., STETLER-STEVENSON, W.G. & LIOTTA, L.A. (1992). Correlation of serum metalloproteinase levels with lung cancer metastasis and response to therapy. *Cancer Res.*, **52**, 4548–4549.

HEMBRY, R.M. & EHRLICH, H.P. (1986). Immunolocalisation of collagenase and tissue inhibitor of metalloproteinases (TIMP) in hypertrophic scar tissue. *Br. J. Dermatol.*, **115**, 409–420.

HEWITT, R.E., LEACH, I.H., POWE, D.G., CLARK, I.M., CAWSTON, T.E. & TURNER, D.R. (1991). Distribution of collagenase and tissue inhibitor of metalloproteinases (TIMP) in colorectal tumours. *Int. J. Cancer*, **49**, 666–672.

LIOTTA, L.A., SHEEG, P.S. & STETLER-STEVENSON, W.G. (1991). Cancer metastasis and angiogenesis: an imbalance of positive and negative regulation. *Cell*, **64**, 327–336.

LU, X., LEVY, M., WEINSTEIN, I.B. & SANTELLA, R.M. (1991). Immunological quantitation of levels of tissue inhibitor of metalloproteinase-1 in human colon cancer. *Cancer Res.*, **51**, 6231–6235.

MULLER, D., WOLF, C., ABECASSIS, J., MILLON, R., ENGELMANN, A., BRONNER, G., ROUYER, N., RIO, M.C., EBER, M., METHLIN, G., CHAMBON, P. & BASSETT, P. (1993). Increased stromelysin-3 gene expression is associated with increased local invasiveness in head and neck squamous cell carcinomas. *Cancer Res.*, **5**, 165–169.

MURPHY, G., COCKETT, M.I., STEPHENS, P.E., SMITH, B.J. & DOCHERTY, A.J.P. (1987). Stromelysin is an activator of procollagenase. A study with natural and recombinant enzymes. *Biochem. J.*, **248**, 265–268.

MURPHY, G., KOKLITIS, P. & CARNE, A.F. (1989). Dissociation of tissue inhibitor of metalloproteinase (TIMP) from enzyme complexes yields fully active inhibitor. *Biochem. J.*, **261**, 1031–1034.

OTANI, Y. (1990). The collagenase activities, interstitial collagenase and type IV collagenase, in human stomach cancer: with special reference to local spreading and lymph node metastasis. *Keio J. Med.*, **39**, 159–167.

POULSOM, R., PIGNATELLI, M., STETLER-STEVENSON, W.G., LIOTTA, L.A., WRIGHT, P.A., JEFFREY, R.E., LONGCROFT, J.A. & STAMP, G.W. (1992). Stromal expression of 72 kDa type-IV collagenase (MMP-2) and TIMP-2 messenger RNAs in colorectal neoplasia. *Am. J. Pathol.*, **141**, 389-396.

PYKE, C., RALFKIAER, E., HUHTALA, P., HURSKAINEN, T., DANO, K. & TRYGGVASON, K. (1992). Localisation of messenger RNA for Mr 72,000 and 92,000 type IV collagenases in human skin cancer by *in situ* hybridization. *Cancer Res.*, **52**, 1336-1341.

STEARNS, M.E. & WANG, M. (1993). Type IV collagenase (Mr 72,000) expression in human prostate: benign and malignant tissue. *Cancer Res.*, **53**, 878-883.

STETLER-STEVENSON, W.G., BROWN, P.D., ONISTO, M., LEVY, A.T. & LIOTTA, L.A. (1990). Tissue inhibitor of metalloproteinases-2 (TIMP-2) mRNA expression in tumour cell lines and human tumour tissues. *J. Biol. Chem.*, **265**, 13933-13938.

SUGARBAKER, E.V. (1981). Patterns of metastasis in human malignancies. *Cancer Biol. Rev.*, **2**, 235-278.

TALHOUK, R.S., BISSELL, M.J. & WERB, Z. (1992). Coordinated expression of extracellular matrix-degrading proteinases and their inhibitors regulates mammary epithelial function during involution. *J. Cell Biol.*, **118**, 1271-1282.

TORTI, F.M. (1983). Response criteria in urologic malignancies: recent results. *Cancer Res.*, **85**, 50-57.

UICC (1978). *TNM. Classification of Malignant Tumors*, 3rd edn, Harmer, M.H. (ed.). UICC: Geneva.

URBANSKI, S.J., EDWARDS, D.R., WATSON, A., WITKIEWICZ, H. & KOSSAKOWSKA, A.E. (1992). Expression of metalloproteinases and their inhibitors in primary pulmonary carcinomas. *Br. J. Cancer*, **66**, 1188-1194.

WAGNER, S.N., RUHRI, C. & KUNTH, K. (1992). Expression of stromelysin 3 in the stromal elements of human basal cell carcinoma. *Diagn. Mol. Pathol.*, **1**, 200-205.

WALAKOVITS, L.A., MOORE, V.L., BHARDWAJ, J.N., GALLICK, G.S. & LARK, M.W. (1991). Detection of stromelysin and collagenase in synovial fluid from patients with rheumatoid arthritis and post-traumatic knee injury. *Arthritis Rheumatism*, **35**, 35-42.

WAXMAN, J., SANDOW, J., THOMAS, H., JAMES, N. & WILLIAMS, G. (1989). A pharmacological evaluation of a new 3-month depot preparation of buserelin for prostatic cancer. *Cancer Chemother. Pharmacol.*, **25**, 219-220.

WAXMAN, J. & WASAN, H. (1992). The architecture of cancer. *Br. Med. J.*, **305**, 1306-1307.

ZUCKER, S., WIEMAN, J., LYSIK, R.M., INHOF, B., NAGASE, H., RAMAMURTHY, N., LIOTTA, L.A. & GOLUB, L.B. (1989). Gelatin degrading type IV collagenase isolated from human small lung cancer. *Invasion Metastasis*, **9**, 167-181.